# Evaluating Uighur literary translation: A comparative study of ChatGPT, Google Translate, and Bing Translator

**DOI:** 10.1371/journal.pone.0335261

**Published:** 2025-10-23

**Authors:** Qiufen Wang

**Affiliations:** School of Languages, Literacies and Translation, Universiti Sains Malaysia, Penang, Malaysia; Wuhan University of Engineering Science, Wuhan, China; Education University of Hong Kong, HONG KONG

## Abstract

This study compares generative artificial intelligence (GenAI) and neural machine translation (NMT) systems in translating Uighur literary text (قۇتادغۇ بىلىك)into English. Two NMT systems, Google Translate and Bing Translator, were evaluated alongside ChatGPT, a GenAI large language model, under two prompt strategies. Translation quality was assessed through automatic metrics (BLEU, ROUGE-N/L, METEOR, and BERT-based semantic similarity), automated error counts (grammar, spelling, style), and expert ratings across four dimensions. Qualitative examples of culturally sensitive excerpts were also examined to illustrate success and failure cases. Results show that ChatGPT, especially with a concise instruction prompt, generally outperforms NMT systems in semantic accuracy, fluency, and cultural adequacy. Bing Translator produced the highest number of errors, particularly spelling mistakes, while Google Translate demonstrated more stable but moderate performance. Statistical testing and expert evaluations supported these patterns, and case analyses revealed how NMT outputs often distorted meaning through polarity reversal and semantic shifts. The findings highlight prompt engineering as a key factor for improving GenAI-based literary translation while recognizing the complementary strengths of GenAI adaptability and NMT stability. Future research should expand language and system coverage and examine the role of human post-editing in enhancing translation quality.

## Introduction

The field of literary translation presents unique challenges, particularly when translating texts from languages rich in cultural nuance and idiomatic expressions, such as Uighur [1]. The nuances embedded in Uighur literature often require more than a literal translation to convey the original’s stylistic and cultural depth, which machine translators (MTs) frequently fail to capture [2]. The dominance of English in academic and literary publishing exacerbates these challenges, as non-English works must be translated with a high degree of accuracy and readability to reach a wider audience [3,4].

With the rapid advancement of neural machine translation (NMT) technologies, tools like Google Translate, Bing Translator, and OpenAI’s ChatGPT have become more accessible and are increasingly employed for everyday translation tasks [5–7]. These tools are built on complex neural network architectures that have significantly improved general translation quality over the past decade [8,9]. However, their effectiveness in translating literary texts, particularly from less commonly translated languages like Uighur, remains underexplored [10]. Studies have shown that while NMTs can handle straightforward texts with some success, they struggle with literary materials that contain layered meanings and stylistic elements [11–13].

Moreover, the application of varying prompts in translation requests represents a novel area of exploration that could potentially enhance the performance of MTs like ChatGPT [14]. Initial research suggests that customized prompts may direct these tools to better handle complex translation tasks by adjusting their output to align more closely with the stylistic and contextual demands of the source text [15,16].

By evaluating translations across five established metrics and analyzing linguistic errors through automated tools, this research provides an empirical assessment of the strengths and limitations of three major MT systems. It also offers insight into the potential of prompt engineering as a practical strategy to enhance literary translation quality in AI-driven tools. In doing so, the study contributes to the broader field of machine-assisted literary translation and supports ongoing efforts to increase the accessibility and global visibility of Uighur literary heritage.

## Literature review

### Machine translation in low-resource languages

Machine translation in low-resource languages has long been recognized as a critical challenge in computational linguistics [17]. Compared with high-resource languages such as English or Chinese, low-resource languages like Uighur, Tibetan, and Mongolian lack parallel corpora, standardized orthography, and digital linguistic resources [18–20]. This scarcity of resources significantly hinders the performance of neural machine translation systems, leading to poor translation accuracy. For some extremely low-resource pairs, BLEU scores remain at single-digit levels—for instance, English to Uyghur baselines achieve only around 0.5–0.7 BLEU [21].

To address these limitations, researchers have proposed a range of strategies. Conventional methods such as segmentation strategies, data augmentation, and multilingual transfer learning have shown potential in mitigating the impact of limited data [22]. Building on these approaches, Jin [23] introduced a semantic word replacement method combined with neural grammar correction to expand bilingual corpora, achieving an improvement of up to 3.06 BLEU. Similarly, Deng and Wang [24] demonstrated that leveraging syntactic structures from source language data, coupled with pre-training strategies, can enhance translation quality, yielding a 1.32 BLEU increase in Chinese–Thai translation tasks. These studies confirm that targeted linguistic and data-driven enhancements can significantly improve performance in low-resource settings.

Recent research has further advanced this field by exploring federated and cross-lingual optimization techniques. Agyei et al. [25] proposed a Cross-Lingual Optimization Framework (CLOF) that dynamically balances gradient aggregation between high-resource and low-resource languages in federated learning. Applied to English–Twi translation, this framework boosted SpBLEU scores from 2.16% to 71.30% after fine-tuning, while also reducing word error rate. Such results illustrate the promise of federated fine-tuning of pretrained multilingual transformers (e.g., mT5) and parameter-efficient adaptation methods (e.g., LoRA, cross-attention adaptation) for scaling MT to a broader range of underrepresented languages.

In addition to improving surface-level accuracy, scholars have also begun to examine whether machine translation preserves higher-level semantic and affective features. Reichel and Benko [26] investigated sentiment transfer in Slovak–English translation using movie subtitles. Their findings revealed a strong correlation between human and machine-translated texts (r = 0.73 overall, increasing to r = 0.86 when neutral sentiment was excluded), showing that sentiment can be reliably preserved through translation. A comparative evaluation with OpenAI’s GPT model reported a slightly lower correlation (r = 0.72), suggesting that while end-to-end large language models provide a convenient alternative, the pipeline approach—translation followed by sentiment analysis in a high-resource language—remains more precise. Importantly, the study also noted challenges in translating humor, irony, and vulgarisms, which often distorted sentiment in comedy subtitles.

Taken together, these findings highlight both the persistent limitations and emerging solutions for machine translation in low-resource languages. While data scarcity and linguistic complexity continue to hinder translation accuracy, advances in data augmentation, syntactic modeling, federated learning, and sentiment preservation indicate promising directions for building more robust and culturally sensitive systems.

### Machine translation of literary texts

The cultural and ideological dimensions of literary translation are central to its evaluation [27]. Scholars continue to debate whether machine-assisted translation can achieve adequacy (faithful representation of meaning) without sacrificing fluency (target-language readability). Recent computational research has shown that the relationship between these two dimensions is more complex than previously assumed. Lim et al. [28] demonstrate that while accuracy and fluency may appear positively correlated at the corpus level, they often trade off against each other at the level of individual segments, a phenomenon explained as Simpson’s paradox. In literary contexts, this paradox is especially salient: strict fidelity to the source may yield stilted renderings, whereas prioritizing fluency risks erasing cultural and ideological nuance.

Beyond these textual properties, researchers have explored how readers perceive translation quality. Martindale et al. [29] introduced the concept of believability—a monolingual reader’s perception that a translation plausibly conveys the source meaning. Their findings reveal that believability aligns more with fluency than adequacy, with 20–25% of outputs across Arabic, Farsi, and Korean judged as “believable but inadequate.” Such fluently misleading translations pose a serious challenge for literary texts, where stylistic plausibility can disguise distortions of cultural or ideological meaning. This highlights the need for evaluation frameworks that consider accuracy, fluency, and believability as distinct yet interrelated dimensions.

Idiomatic and figurative expressions further complicate literary machine translation. Obeidat et al. [30] examined 155 English idioms translated into Arabic by Google Translate, ChatGPT, and Gemini. The results showed that Google Translate overwhelmingly relied on literal translation (77%), often producing nonsensical outputs, while ChatGPT displayed more paraphrasing (53%). Gemini most frequently employed sense-based strategies (63%) and achieved the highest proportion of idiomatic-to-idiomatic renderings (16%). Despite these improvements, all systems struggled with non-compositional idioms such as “speak of the devil,” which require cultural and pragmatic competence beyond lexical substitution. These findings confirm that idiomatic language remains a persistent challenge, limiting the adequacy of literary MT.

An equally pressing issue arises in the translation of politically and ideologically charged texts, where meaning extends beyond style to cultural and historical identity. Obeidat and Jaradat [31] analyzed AI translations of Ghassan Kanafani’s short story Until We Return using Berman’s twelve deforming tendencies. Their study found that both Google Translate and ChatGPT exhibited extensive distortions: rationalization accounted for 36% of errors, qualitative impoverishment for 32% (Google) and 26% (ChatGPT), and destruction of rhythms for around 10% in both systems. These tendencies led to mistranslations of gendered pronouns, weakened metaphors, and disrupted narrative flow, ultimately compromising the cultural resonance and political force of the text. The authors conclude that while AI tools may assist in processing resistance literature, human translators remain indispensable for preserving its ideological depth and cultural authenticity.

Taken together, these studies illustrate the complex interplay of accuracy, fluency, believability, and cultural representation in the machine translation of literary texts. Advances in neural and generative models have improved the handling of idiomatic expressions and stylistic plausibility, yet significant limitations remain, especially in preserving cultural and ideological dimensions. This underscores the necessity of integrating human expertise with computational approaches to ensure that translations of literary works not only function linguistically but also honor the aesthetic, cultural, and political essence of the original.

### ChatGPT and prompt engineering for translation

Google Translate and Bing Translator are neural machine translation tools trained on large-scale bilingual corpora with encoder–decoder architectures, and their outputs are relatively stable but limited in adapting to low-resource or stylistically complex texts [22]. By contrast, ChatGPT is a generative AI large language model designed for multiple natural language tasks, including but not limited to translation [32]. Unlike corpus-driven NMT, ChatGPT relies on probabilistic reasoning over vast general-purpose data and is therefore prompt-driven, offering flexibility and stylistic adaptation but also variability and inconsistency.

The rise of LLMs has made prompt engineering a key factor in translation studies, allowing users to shape outputs without model fine-tuning. He [33] tested different prompt types—basic instructions, translation briefs, and persona-based prompts—and found that the “translator persona” yielded the most natural translations. This suggests that conventional professional briefs may not transfer seamlessly to human–machine interaction, while persona-driven strategies align more closely with generative models.

Building on this, Yamada [14] incorporated translation specifications from ISO 17100 into prompts. Parameters such as communicative purpose and audience improved ChatGPT’s handling of idiomatic expressions and cultural references. For example, when translating the Japanese idiom *we are friends who ate rice from the same pot*, purpose-driven prompts guided the system to produce the idiomatic English equivalent *we have been through thick and thin together*. Such findings show that prompt engineering can approximate pre-production decisions typically made by human translators.

Gao et al. [15] further evaluated prompt designs by treating ChatGPT as a black box. Enriched prompts containing task information, domain cues, or part-of-speech tags consistently outperformed simple commands, and in some cases even surpassed commercial systems like Google Translate and DeepL. However, overly complex prompts sometimes reduced performance, indicating the need for balance between specificity and simplicity.

Finally, Jiang et al. [34] compared ChatGPT with Google Translate, Microsoft Translator, and DeepL using both automatic metrics and human judgments. Zero-shot prompts provided competitive baselines, but one-shot and context-aware prompts produced substantial improvements in clarity, style, and cultural sensitivity. Interestingly, these enhancements were not fully captured by traditional metrics such as BLEU, but were reflected in semantic-oriented measures like BERTScore and COMET, which aligned more closely with human evaluations.

Taken together, these studies underscore that prompt engineering enhances aspects of translation quality—such as pragmatic appropriateness, cultural adaptation, and stylistic coherence—that are often overlooked by automatic metrics. While NMT systems remain stable and efficient, ChatGPT’s flexibility makes it particularly responsive to well-designed prompts. This distinction reinforces the importance of considering prompt design not merely as a technical adjustment, but as a central variable in evaluating and improving LLM-based translation.

## Methods

This study adopts a primarily quantitative, product-oriented design, complemented by qualitative analysis. Quantitative evaluation was carried out using automatic metrics, statistical testing, and error counts, while qualitative examples of culturally and stylistically sensitive translations were incorporated to provide deeper insight into how errors manifest in practice.

### Selection of Uighur literature and translation systems

The first ten chapters of the Uyghur original text قۇتادغۇ بىلىك, published by Xinjiang People’s Publishing House, were selected as the source text [35], and its English translation, Wisdom of Royal Glory translated by Robert Dankoff, was used as reference text [36]. This work, through the dialogue between four fictional symbolic characters, deeply and meticulously praises God and the Prophet, sings praises to wise monarchs, and advises rulers to be fair, wise, and contented. It also analyzes and evaluates the practical role of various industries at that time. This literary work was selected because it contains rich Uyghur cultural elements, and the novel is the first large-scale literary work written in pure Uyghur language, and successfully introduced the Aruzi rhythm, which has a profound impact on the development of Uyghur poetry in later generations [37,38]. Its cultural and stylistic richness makes it an ideal case for evaluating translation systems. Although “قۇتادغۇ بىلىك” consists of 82 chapters in total, this study focused on the first ten chapters. Each chapter is a long poetic unit, unlike Chinese classical quatrains that contain only a few lines, and thus provides substantial material for evaluation. Selecting ten chapters ensured both feasibility for detailed analysis and sufficient representativeness to capture the work’s cultural and stylistic richness.

For comparison, two neural machine translation (NMT) systems—Google Translate and Bing Translator—and one generative AI large language model (LLM), ChatGPT, were employed. Google Translate and Bing Translator are corpus-driven NMT systems trained on large-scale bilingual data using encoder–decoder architectures. By contrast, ChatGPT is a general-purpose LLM developed by OpenAI, with translation as only one of its many applications. In this study, ChatGPT (GPT-4o, accessed via the official ChatGPT web interface) was used, as this detail is important for reproducibility, given that different versions and access methods may yield different results.

These three systems were selected for specific reasons. Google Translate and Bing Translator were included because they are among the few commercial MT tools that support Uyghur–English translation, while other platforms such as DeepL currently do not. ChatGPT was included not as a traditional MT system but as a widely used LLM, in order to examine how a prompt-driven generative model performs on low-resource literary translation compared with dedicated NMT systems. Although other LLMs (e.g., Gemini, DeepSeek, Cohere, LLaMA) are available, ChatGPT was prioritized because of its accessibility, widespread use among both researchers and the general public, and its established baseline performance reported in previous translation studies.

### Selection of metrics to evaluate the accuracy of translation

This study employed a set of well-established metrics to assess the accuracy, fluency, and stylistic fidelity of machine-translated texts from Uyghur to English. These metrics are essential for evaluating how effectively translation tools handle literary texts, where contextual and stylistic nuances are as important as literal correctness.

One widely used metric is BLEU (Bilingual Evaluation Understudy Score), which measures translation quality by comparing n-gram matches between the machine output and reference translations [39]. N-grams are sequences of words, such as unigrams (single words) or bigrams (pairs of words) [40]. BLEU calculates the proportion of matching n-grams while adjusting for translation length to prevent bias toward overly short outputs [41]. Due to its strong correlation with human judgment, BLEU remains a standard in translation evaluation [42].

Another important metric is ROUGE (Recall-Oriented Understudy for Gisting Evaluation), which evaluates translation quality based on n-gram overlap with reference texts [43]. Unlike BLEU, ROUGE emphasizes recall, measuring how much of the reference content is captured in the translation [44]. ROUGE includes multiple variants, such as ROUGE-N, which assesses n-gram recall, and ROUGE-L, which examines the longest common subsequence of words between the translation and reference [45]. These variants help evaluate both content coverage and structural fluency.

For a more nuanced assessment, METEOR was also used. Unlike BLEU and ROUGE, METEOR incorporates semantic matching by considering synonyms, paraphrases, and word stems [46]. This allows for a more flexible evaluation, where translations with different but semantically equivalent wording can still receive high scores.

In addition, BERT-based semantic similarity was employed, which uses deep learning to compare contextual embeddings of translated and reference texts [47]. BERT (Bidirectional Encoder Representations from Transformers) captures meaning beyond surface-level word matching, making it particularly useful for assessing semantic accuracy in literary translations [48].

To implement these metrics, Python libraries such as NLTK for ROUGE and sacreBLEU for BLEU scoring were applied. The results were visualized using matplotlib and seaborn to facilitate clear comparisons across different translation tools (see Table 1). This approach ensures transparency and reproducibility, aligning with academic research standards.

**Table 1:** Key metrics for evaluating translation accuracy.

Metric	Description	Focus Area	Formula Used
**BLEU**	Measures the precision of n-grams between the translated text and one or more reference texts.	Lexical accuracy	$BLEU=BP*exp\left(\sum \left(\text{log}\left(p_{n}\right)/N\right)\right)$ *[pn*: n-gram precision; *BP*: Penalty for short translations]
**ROUGE-N**	Evaluates the overlap of n-grams between the machine-generated text and the reference texts.	Content recall and fluency	ROUGE−N = Count(matching n−grams/ Count(n−grams in reference)
**ROUGE-L**	Measures the longest common subsequence between the translation and the reference text.	Fluency and word order	ROUGE−L = Length(LCS/ Length(reference)
**METEOR**	Aligns the translation with the reference for semantic and syntactic accuracy using stemming and synonyms.	Semantic and syntactic accuracy	$METEOR=F_{\text{1}\text{2}}\;*\;(\text{1}-Penalty)$ [*F₁₂*: Harmonic mean of precision/recall; *Penalty*: For poor alignment]
**BERT**	Uses BERT embeddings to assess semantic similarity between translated and reference texts.	Semantic fidelity	BERT−Score = Cosine( Embedding(translation), Embedding(reference) )

In addition to automatic evaluation, expert human ratings were incorporated to strengthen the reliability of the findings. Two professional translators of Uyghur ethnicity, each with over eight years of experience and fluent in Uyghur, Chinese, and English, independently evaluated the translations. Following the evaluation framework used in Gao et al. [49], the experts rated each translation on four dimensions—Fidelity, Fluency, Cultural Fidelity & Style, and Machine Translation Style—using a 5-point Likert scale (1 = very poor, 5 = excellent). The relatively small sample of two experts was chosen in line with prior studies adopting limited but specialized rater groups for in-depth literary translation assessment, while balancing feasibility and reliability. To ensure the validity of their judgments, inter-rater consistency was examined before aggregating the scores for further analysis.

### Selection of translation errors

This study uses LanguageTool to identify errors in machine translations from Uighur to English. LanguageTool is a grammar checking tool that finds mistakes in texts [50]. We apply it to translations to find problems with grammar, word choice, and style.

LanguageTool checks several types of errors [51]. First, it finds grammar mistakes. These include wrong verb forms and incorrect sentence structures. For example, it can detect when a verb does not match its subject. Second, it identifies spelling errors. This helps find words that are spelled wrong or used incorrectly. Third, it checks punctuation. This includes missing commas or wrong quotation marks. Fourth, it finds style issues. These are phrases that sound unnatural or awkward.

The tool works by comparing text against rules [52]. It has rules for English grammar and usage. When text breaks these rules, it flags the error [53].

We categorize the errors into groups. The main groups are grammar, spelling, and style. Punctuation-related issues were excluded from this analysis due to inconsistent detection and limited relevance to the core objectives of literary translation evaluation.

For each translation, errors were recorded at the chapter level and classified by type. The number of occurrences for each error type was counted per chapter, per tool. This approach enabled a comparative analysis of the frequency and distribution of linguistic errors across translation systems. The tool was run in a fully automated manner to ensure reproducibility and minimize subjectivity in error identification.

Although the error detection process was primarily automated, representative samples were manually reviewed to ensure the tool’s reliability. This review helped confirm that the errors identified were linguistically valid and that no major categories of errors were systematically missed.

This approach helps evaluate translation systems objectively. It finds actual errors that readers would notice. The results can guide improvements in machine translation. They show specific areas where systems need work. This is valuable for making better Uighur to English translations.

### Statistical analysis

A statistical analysis was conducted of translations generated by three machine translation tools—ChatGPT, Bing Translator, and Google Translate—using the first ten chapters of the Uighur literary قۇتادغۇ بىلىك (Wisdom of Royal Glory) as source material. The analysis aimed to assess translation performance in terms of accuracy, fluency, and semantic preservation, as well as to investigate the influence of prompt variation on translation quality. A particular focus was placed on evaluating whether tailored prompts could reduce stylistic features commonly associated with machine-generated translations. To explore this, two distinct prompt formulations were used with ChatGPT. The first prompt was a straightforward instruction: “Please provide the English translation for the following material.” The second prompt was more interpretive, instructing: “The following are Uighur poetry, please interpret their meaning first and translate them into English, preserving the poetic form and style.” The use of prompt variation is grounded in recent literature, which demonstrates that carefully crafted instructions can significantly influence the quality of ChatGPT’s translations, especially in complex tasks involving literary or poetic content [54,55]. These studies support the idea that prompt engineering can serve as a key variable in enhancing ChatGPT’s capacity to balance literal accuracy with stylistic and cultural fidelity.

To quantify translation performance, four automatic evaluation metrics were employed: BLEU, ROUGE, METEOR, and BERT-based semantic similarity. All scores were computed for each chapter across all four tools and prompt conditions. Given the small sample size and potential deviations from normality, the results were summarized using medians and interquartile ranges (IQRs), which are more robust for non-parametric data. For group comparisons, the Kruskal-Wallis H test was used. Where significant differences were found, Dunn’s post-hoc test with Sidak correction was conducted to examine pairwise contrasts.

In addition to metric-based analysis, translation errors were examined using LanguageTool, a Python library that automatically detects errors in grammar, spelling, punctuation, and style. Errors were recorded and categorized by type and chapter, and frequency counts were compiled for each translation system. To identify significant differences in error patterns across systems, one-way ANOVA tests were conducted on the total counts of grammar, spelling, and style errors. Where statistical significance was detected, Tukey-style post-hoc tests were used to explore pairwise differences.

All statistical analyses **w**ere carried out using Python, including libraries such as SciPy, NumPy, Pandas, and Seaborn for data processing and visualization. For machine translation evaluation, NLTK was used for ROUGE and sacreBLEU for BLEU scoring. Results were illustrated using tables and graphs to facilitate interpretation and support comparison across translation tools and prompt designs.

This methodological framework enabled a comprehensive comparison of translation performance across systems and configurations. The combination of evaluation metrics, statistical testing, and detailed error classification provided robust evidence of each system’s strengths and limitations in handling complex literary texts. These findings offer practical insights for improving prompt engineering and refining machine translation outputs in the context of low-resource and high-literary-value languages such as Uyghur.

### Ethical considerations

This study did not involve human participants or the collection of personally identifiable information, as the primary focus was on machine-generated translations. The expert evaluations were conducted by two professional translators who assessed the quality of the translations in their professional capacity. Their participation was fully voluntary and based on informed consent. Given that no personal or sensitive data were collected, formal ethical approval was not required for this study.

## Results

### Comparative performance of different translation tools

First, the BLEU scores for each tool show that ChatGPT with Prompt 1 generally produces higher scores than its alternatives, although it does not significantly outperform Google Translate or Bing Translator in all chapters. As shown in Table 2, ChatGPT with Prompt 1 has a notably higher BLEU score in Chapter 1 (0.0333) compared to the other tools, but its performance decreases in subsequent chapters. This trend can be observed consistently across all chapters, indicating that BLEU scores for ChatGPT with Prompt 1 are more variable than those for Google Translate and Bing Translator. This finding suggests that, while ChatGPT can produce good results under certain conditions, it is not always the most reliable tool across a range of evaluation metrics. On the other hand, Google Translate and Bing Translator tend to provide more stable BLEU scores across chapters, but without a significant advantage over ChatGPT.

**Table 2:** BLEU Scores for Translations by ChatGPT (with Prompts 1 and 2), Google Translate, and Bing Translator across Chapters.

Chapter	BLEU_ChatGPT with Prompt 1	BLEU_ChatGPT with Prompt 2	BLEU_Google Translate	BLEU_Bing Translator
1	0.0333	0.0049	0.0215	0.0075
2	0.0178	0.0070	0.0139	0.0093
3	0.0100	0.0075	0.0298	0.0162
4	0.0067	0.0024	0.0177	0.0167
5	0.0163	0.0086	0.0206	0.0094
6	0.0098	0.0097	0.0168	0.0097
7	0.0099	0.0121	0.0218	0.0128
8	0.0111	0.0053	0.0180	0.0125
9	0.0067	0.0039	0.0196	0.0253
10	0.0137	0.0017	0.0234	0.0136

Moving to ROUGE-N scores, Table 3 reveals that ChatGPT with Prompt 1 consistently scores higher than other tools in most chapters, particularly in Chapter 7, where it reaches 0.4828. This indicates that, at least in some cases, ChatGPT with Prompt 1 is better at preserving content during translation, especially with respect to the use of relevant phrases. However, like the BLEU scores, its performance varies across different chapters. Google Translate and Bing Translator, while performing well, show somewhat lower scores across most chapters, suggesting that they may be less effective at capturing meaningful content or phrase structure when compared to ChatGPT with Prompt 1.

**Table 3:** ROUGE-N Scores for Translations by ChatGPT (with Prompts 1 and 2), Google Translate, and Bing Translator across Chapters.

Chapter	ROUGE_N_ChatGPT with Prompt 1	ROUGE_N_ChatGPT with Prompt 2	ROUGE_N_Google Translate	ROUGE_N_Bing Translator
1	0.4693	0.3442	0.4132	0.3473
2	0.4105	0.4070	0.3063	0.2716
3	0.3648	0.3429	0.3193	0.3063
4	0.4264	0.3168	0.3919	0.3534
5	0.3618	0.3281	0.3116	0.2997
6	0.3183	0.3326	0.3694	0.2907
7	0.4828	0.4429	0.4604	0.4073
8	0.4402	0.3072	0.4119	0.3930
9	0.4351	0.3284	0.4493	0.4154
10	0.4202	0.2880	0.4487	0.4266

When analyzing ROUGE-L scores, presented in Table 4, it is clear that ChatGPT with Prompt 1 again performs well, with scores higher than those of Google Translate and Bing Translator in several chapters, particularly in Chapter 1, where the score reaches 0.2329. However, the difference in scores is more modest here, suggesting that the translation tools are comparable in terms of their ability to maintain the linguistic integrity and fluency of the translated text. This is in line with the findings from the ROUGE-N metric, where the tools exhibit similar trends in performance across chapters.

**Table 4:** ROUGE-L Scores for Translations by ChatGPT (with Prompts 1 and 2), Google Translate, and Bing Translator across Chapters.

Chapter	ROUGE_L_ChatGPT with Prompt 1	ROUGE_L_ChatGPT with Prompt 2	ROUGE_L_Google Translate	ROUGE_L_Bing Translator
1	0.2329	0.1500	0.2048	0.1541
2	0.2093	0.2153	0.1531	0.1552
3	0.2066	0.1633	0.2129	0.1707
4	0.1738	0.1434	0.2013	0.1705
5	0.1757	0.1470	0.1573	0.1485
6	0.1505	0.1600	0.1783	0.1475
7	0.1911	0.1667	0.2000	0.1606
8	0.1781	0.1322	0.1956	0.1870
9	0.1521	0.1333	0.2108	0.1991
10	0.1469	0.1181	0.1898	0.1649

METEOR scores, as shown in Table 5, support similar conclusions. ChatGPT with Prompt 1 generally outperforms the other tools in terms of semantic adequacy and fluency, particularly in Chapters 1, 2, and 7. However, the differences between the tools are relatively small, suggesting that while ChatGPT with Prompt 1 performs well, Google Translate and Bing Translator are competitive in terms of their ability to capture meaning and fluency. The highest METEOR score for ChatGPT with Prompt 1 is 0.2456 in Chapter 7, indicating its ability to preserve meaning in certain contexts.

**Table 5:** METEOR Scores for Translations by ChatGPT (with Prompts 1 and 2), Google Translate, and Bing Translator across Chapters.

Chapter	METEOR_ChatGPT with Prompt 1	METEOR_ChatGPT with Prompt 2	METEOR_Google Translate	METEOR_Bing Translator
1	0.2449	0.1588	0.2093	0.1605
2	0.2017	0.1987	0.1336	0.1237
3	0.1775	0.1891	0.1313	0.1557
4	0.1941	0.1175	0.2030	0.1622
5	0.1735	0.1658	0.1376	0.1489
6	0.1826	0.1687	0.1883	0.1408
7	0.2456	0.2005	0.2468	0.1982
8	0.2169	0.1358	0.2269	0.1861
9	0.2153	0.1371	0.2340	0.2026
10	0.1992	0.1032	0.2407	0.1941

Finally, the BERT scores, as shown in Table 6, further highlight ChatGPT’s superiority in certain chapters, particularly in Chapter 7, where it scores 0.6243. This suggests that ChatGPT with Prompt 1 can better maintain contextual understanding and semantic meaning in the translation, aligning with the findings from other metrics. Google Translate and Bing Translator show lower but relatively consistent performance, reinforcing the idea that while these tools are reliable, they may not offer the same level of semantic preservation and fluency as ChatGPT with Prompt 1.

**Table 6:** BERT Scores for Translations by ChatGPT (with Prompts 1 and 2), Google Translate, and Bing Translator across Chapters.

Chapter	BERT_ChatGPT with Prompt 1	BERT_ChatGPT with Prompt 2	BERT_Google Translate	BERT_Bing Translator
1	0.6480	0.5945	0.5893	0.5410
2	0.6116	0.5969	0.5611	0.5215
3	0.6134	0.5950	0.5830	0.5525
4	0.5942	0.5819	0.5817	0.5397
5	0.5906	0.5511	0.5677	0.5250
6	0.6029	0.6006	0.6155	0.5394
7	0.6243	0.6096	0.6160	0.5602
8	0.5943	0.5655	0.6044	0.5444
9	0.5918	0.5891	0.6006	0.5508
10	0.5982	0.5757	0.6076	0.5683

**Fig 1:**
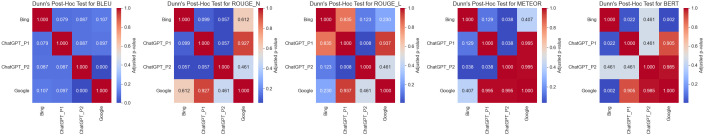
Post-Hoc Analysis of Translation Tools Across Multiple Evaluation Metrics.

**Fig 2:**
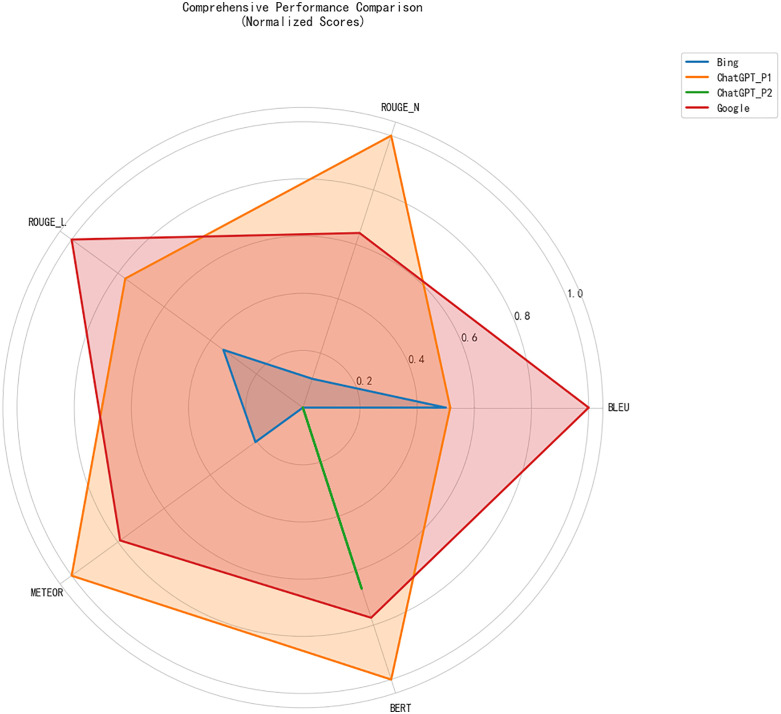
Comprehensive Performance Comparison of Translation Tools Across Five Evaluation Metrics.

In summary, while ChatGPT with Prompt 1 performs well across most metrics, it does not always outshine Google Translate or Bing Translator, especially in terms of BLEU and ROUGE-L scores. The comparison between the tools shows that each has its strengths and weaknesses, with ChatGPT with Prompt 1 performing well in terms of content preservation and semantic fluency, while Google Translate and Bing Translator are more stable in terms of score consistency across chapters. These findings are visualized in Fig 1, which shows the post-hoc significance analysis of translation tools across the evaluation metrics. Fig 2 provides a comprehensive performance comparison of translation tools across the five metrics, further highlighting the relative strengths of each tool.

### Impact of different prompts on ChatGPT’s translation performance

The Wilcoxon Signed-Rank Test results, presented in Table 7, demonstrate statistically significant differences between the two prompts across all metrics. Prompt 1 consistently performs better than Prompt 2, with effect sizes indicating large differences (r = 0.72 for BLEU, 0.75 for ROUGE-N, and 0.66 for METEOR). The p-values for all metrics are less than 0.01, signifying that the differences in performance are highly significant. This suggests that Prompt 1 encourages ChatGPT to produce translations closer to the original text, reflecting higher accuracy in terms of phrase-level preservation and semantic consistency.

**Table 7:** Wilcoxon Signed-Rank Test Results Comparing ChatGPT Prompt Strategies Across Evaluation Metrics.

Metric	W Statistic	p-value	Effect Size (r)	Median (P1)	Median (P2)
BLEU	23.0	0.0059**	0.72	0.011	0.007
ROUGE-N	25.0	0.0059**	0.75	0.426	0.329
ROUGE-L	21.0	0.0098**	0.68	0.178	0.143
METEOR	20.0	0.0098**	0.66	0.201	0.137
BERT	28.0	0.0020**	0.79	0.613	0.581

*Note.* ** indicates statistical significance after Bonferroni correction (adjusted α = 0.01). Effect sizes (r) are interpreted as: 0.1–0.3 (small), 0.3–0.5 (medium), > 0.5 (large). P1 = Prompt 1, P2 = Prompt 2. All tests two-tailed with N = 10 per group.

The BLEU scores, shown in Table 2, confirm that Prompt 1 consistently outperforms Prompt 2. For instance, Prompt 1 achieves higher BLEU scores in all chapters, with a notable gap in Chapter 1 (0.0333 for Prompt 1) and smaller differences in later chapters. Fig 1 illustrates the post-hoc significance matrix for BLEU scores, showing that the differences between the two prompts are statistically significant in the majority of chapters.

When analyzing ROUGE-N and ROUGE-L scores, the data from Table 3 and Table 4 show that Prompt 1 yields higher scores in several chapters, particularly in Chapters 1, 7, and 9. These results suggest that Prompt 1 better preserves content, such as key phrases and sentence structures, which is crucial for achieving higher ROUGE-N and ROUGE-L scores. Fig 2, which visualizes the post-hoc significance matrix for ROUGE-N, further reinforces that Prompt 1 leads to better phrase-level preservation across different translation tools.

**Fig 3:**
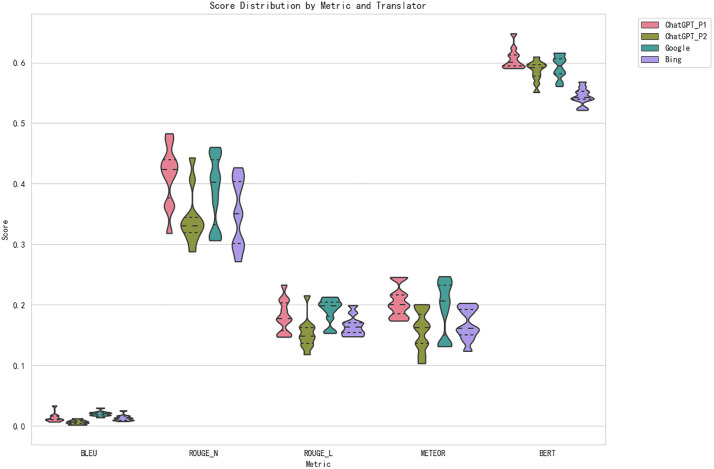
Distribution of Evaluation Scores by Metric and Translation Tool.

In terms of METEOR scores, Table 5 reveals that Prompt 1 consistently produces higher scores compared to Prompt 2, which indicates a better ability of Prompt 1 to capture semantic meaning and fluency. Fig 3, which presents the distribution of evaluation scores by metric and translation tool, demonstrates that Prompt 1 leads to more balanced and consistent scores, further suggesting that it is more effective in preserving the overall context and meaning in the translations.

**Fig 4:**
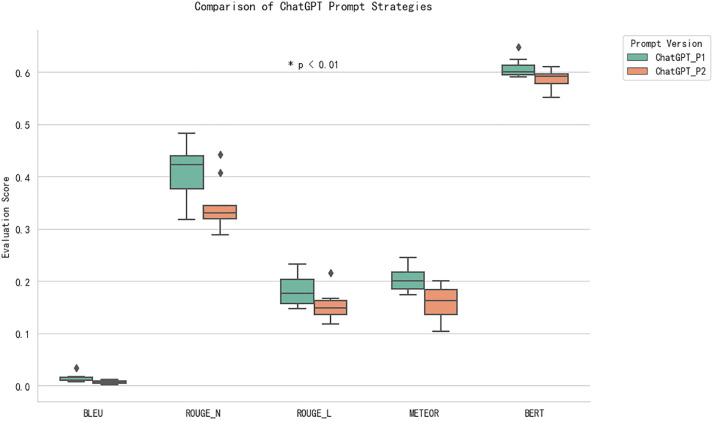
Comparison of ChatGPT Prompt Strategies Across Multiple Evaluation Metrics.

For BERT scores, Table 6 shows that Prompt 1 again performs better than Prompt 2, especially in Chapter 7 (0.6243 for Prompt 1), confirming that Prompt 1 is more successful in maintaining the contextual understanding of the original text. Fig 4 further supports this conclusion, highlighting Prompt 1’s superior performance across most evaluation metrics, especially in terms of semantic preservation, as indicated by its higher BERT-based semantic similarity scores.

### Expert evaluation of translation quality

To ensure the reliability of the human evaluation data, inter-rater agreement between the two experts was assessed using Cohen’s Kappa. As shown in Table 8, quadratic weighted κ values indicated substantial agreement across all four dimensions, with the highest consistency in Fidelity (κ = 0.821) and Machine Translation Style (κ = 0.853), while Cultural Fidelity & Style (κ = 0.752) and Fluency (κ = 0.756) also showed substantial reliability. Overall agreement across all dimensions and systems was substantial (quadratic weighted κ = 0.804; Table 9), confirming that the expert ratings are dependable and suitable for integration with the automatic evaluation metrics.

**Table 8:** Inter-rater reliability by rating dimension (quadratic weighted Cohen’s Kappa).

Dimension	N_pairs	Percent agreement	Kappa (Quadratic)	Interpretation
Fidelity	40	0.525	0.821	Substantial
Fluency	40	0.450	0.756	Substantial
Cultural Fidelity & Style	40	0.400	0.752	Substantial
Machine Translation Style	40	0.600	0.853	Almost perfect

**Table 9:** Overall inter-rater reliability across all dimensions and systems.

N_pairs	Percent agreement	Kappa (Quadratic)	Interpretation
160	0.494	0.804	Substantial

This strong level of agreement supports the robustness of combining automatic metrics with expert judgments, providing complementary perspectives on translation performance. Descriptive statistics of human ratings across systems and dimensions are presented in Table 10. Here, System A refers to ChatGPT with Prompt 1, System B to ChatGPT with Prompt 2, System C to Google Translate, and System D to Bing Translator. The results show that ChatGPT with Prompt 1 (System A) achieved the highest mean and median scores in Fidelity (M = 4.6500), Fluency (M = 4.5500), and Cultural Fidelity & Style (M = 4.5500), while scoring lowest on Machine Translation Style (M = 1.4500), indicating translations that were accurate, fluent, and culturally faithful without exhibiting strong machine-like features. ChatGPT with Prompt 2 (System B) performed moderately across all four dimensions (means between 3.4000–3.4500), reflecting balanced but less distinguished output compared to System A. Google Translate (System C) showed slightly lower scores overall (means between 2.5500–3.1000), with middling performance in Fluency and Cultural Fidelity. Bing Translator (System D) displayed the highest score on Machine Translation Style (M = 4.6000), suggesting outputs strongly resembling machine-generated language, but it performed the worst on Fidelity, Fluency, and Cultural Fidelity & Style (means between 1.8000–2.0000).

**Table 10:** Descriptive statistics by system × dimension (pooled raters).

System	Dimension	N	Mean	SD	Median	Q1	Q3	IQR
A	Cultural Fidelity & Style	20	4.5500	0.5104	5.0000	4.0000	5.0000	1.0000
A	Fidelity	20	4.6500	0.4894	5.0000	4.0000	5.0000	1.0000
A	Fluency	20	4.5500	0.5104	5.0000	4.0000	5.0000	1.0000
A	Machine Translation Style	20	1.4500	0.5104	1.0000	1.0000	2.0000	1.0000
B	Cultural Fidelity & Style	20	3.4500	0.5104	3.0000	3.0000	4.0000	1.0000
B	Fidelity	20	3.4500	0.5104	3.0000	3.0000	4.0000	1.0000
B	Fluency	20	3.4000	0.5026	3.0000	3.0000	4.0000	1.0000
B	Machine Translation Style	20	2.3000	0.4702	2.0000	2.0000	3.0000	1.0000
C	Cultural Fidelity & Style	20	2.5500	0.5104	3.0000	2.0000	3.0000	1.0000
C	Fidelity	20	2.8500	0.5871	3.0000	2.7500	3.0000	0.2500
C	Fluency	20	2.9500	0.5104	3.0000	3.0000	3.0000	0.0000
C	Machine Translation Style	20	3.1000	0.6407	3.0000	3.0000	3.2500	0.2500
D	Cultural Fidelity & Style	20	1.9500	0.5104	2.0000	2.0000	2.0000	0.0000
D	Fidelity	20	1.8000	0.5231	2.0000	1.7500	2.0000	0.2500
D	Fluency	20	2.0000	0.6489	2.0000	2.0000	2.0000	0.0000
D	Machine Translation Style	20	4.6000	0.5026	5.0000	4.0000	5.0000	1.0000

### Translation errors across different tools and prompts

According to Table 11, Bing Translator consistently exhibits a higher number of errors across all categories, particularly in spelling. For instance, in Chapter 4, Bing Translator made a total of 74 spelling errors, the highest among all the translation tools. In comparison, ChatGPT with Prompt 1 and Prompt 2 exhibit significantly fewer errors, especially in grammar. For example, ChatGPT with Prompt 1 made only 3 grammar errors in Chapter 1, and Prompt 2 made no errors at all. Google Translate also produced moderate numbers of errors, but it performed more consistently than Bing Translator, especially in the spelling category. Table 9 further illustrates the descriptive statistics of these errors, including the means and standard deviations for each error type.

**Table 11:** Distribution of Translation Errors by Type and Chapter for Each Machine Translation Tool.

Chapter	Translator	Grammar	Spelling	Style	Total Errors
1	ChatGPT with Prompt 1	3	0	0	3
1	ChatGPT with Prompt 2	0	0	0	0
1	Google Translate	9	0	1	10
1	Bing Translator	8	12	0	20
2	ChatGPT with Prompt 1	0	0	0	0
2	ChatGPT with Prompt 2	2	0	0	2
2	Google Translate	0	0	0	0
2	Bing Translator	5	10	0	15
3	ChatGPT with Prompt 1	0	0	1	1
3	ChatGPT with Prompt 2	0	0	0	0
3	Google Translate	0	1	0	1
3	Bing Translator	3	8	0	11
4	ChatGPT with Prompt 1	1	3	1	5
4	ChatGPT with Prompt 2	0	2	0	2
4	Google Translate	5	6	1	12
4	Bing Translator	12	74	0	86
5	ChatGPT with Prompt 1	0	0	0	0
5	ChatGPT with Prompt 2	0	0	0	0
5	Google Translate	1	0	0	1
5	Bing Translator	5	24	0	29
6	ChatGPT with Prompt 1	0	0	0	0
6	ChatGPT with Prompt 2	0	0	0	0
6	Google Translate	0	0	0	0
6	Bing Translator	1	2	1	4
7	ChatGPT with Prompt 1	0	0	0	0
7	ChatGPT with Prompt 2	0	1	0	1
7	Google Translate	1	1	0	2
7	Bing Translator	6	16	0	22
8	ChatGPT with Prompt 1	1	0	1	2
8	ChatGPT with Prompt 2	1	0	0	1
8	Google Translate	6	0	0	6
8	Bing Translator	14	23	0	37
9	ChatGPT with Prompt 1	2	1	0	3
9	ChatGPT with Prompt 2	2	0	0	2
9	Google Translate	4	1	0	5
9	Bing Translator	8	43	0	51
10	ChatGPT with Prompt 1	0	0	0	0
10	ChatGPT with Prompt 2	0	0	0	0
10	Google Translate	9	2	0	11
10	Bing Translator	13	27	1	41

From the descriptive statistics in Table 12, we observe that Bing Translator has the highest mean number of errors across all categories: 7.5 grammar errors, 23.9 spelling errors, and 31.6 total errors. This is in stark contrast to ChatGPT with Prompt 2, which made fewer errors overall, with a mean of just 0.5 grammar errors and 0.8 total errors. ChatGPT with Prompt 1 also demonstrated relatively low error rates, with a mean of 0.7 grammar errors and 1.4 total errors, indicating that both prompts generally help reduce errors, especially when compared to Bing Translator.

**Table 12:** Descriptive Statistics of Error Types by Translation Tool (Mean±SD).

Translator	Grammar mean	Grammar std	Spelling mean	Spelling std	Style mean	Style std	Total Errors mean	Total Errors std
Bing Translator	7.5	4.35	23.9	21.12	0.2	0.42	31.6	23.92
ChatGPT with Prompt 1	0.7	1.06	0.4	0.97	0.3	0.48	1.4	1.78
ChatGPT with Prompt 2	0.5	0.85	0.3	0.67	0	0	0.8	0.92
Google Translate	3.5	3.63	1.1	1.85	0.2	0.42	4.8	4.73

In addition, a one-way analysis of variance (Table 13) was conducted to determine if there were significant differences in errors between the translation tools. The results show significant differences in grammar and spelling errors, with Bing Translator consistently performing worse than the other tools. Table 14 presents the post-hoc analysis of error variations, highlighting that Bing Translator made significantly more errors compared to ChatGPT with Prompt 1, ChatGPT with Prompt 2, and Google Translate.

**Table 13:** One-way Analysis of Variance for Grammar, Spelling, and Style Errors Among Translation Tools.

Error Type	F-value	p-value	Significant
Grammar	12.577	0	Yes
Spelling	12.051	0	Yes
Style	1.075	0.3717	No
Total Errors	14.52	0	Yes

**Table 14:** Post-hoc Analysis of Translation Error Variations Among Four MT System.

ErrorType	Group1	Group2	MeanDiff	p-value	Sig
Grammar	Bing Translator	ChatGPT with Prompt 1	−6.8	4.41E-05	TRUE
Grammar	Bing Translator	ChatGPT with Prompt 2	−7	2.76E-05	TRUE
Grammar	Bing Translator	Google Translate	−4	0.020235	TRUE
Grammar	ChatGPT with Prompt 1	ChatGPT with Prompt 2	−0.2	0.998687	FALSE
Grammar	ChatGPT with Prompt 1	Google Translate	2.8	0.157317	FALSE
Grammar	ChatGPT with Prompt 2	Google Translate	3	0.11639	FALSE
Spelling	Bing Translator	ChatGPT with Prompt 1	−23.5	1.00E-04	TRUE
Spelling	Bing Translator	ChatGPT with Prompt 2	−23.6	9.38E-05	TRUE
Spelling	Bing Translator	Google Translate	−22.8	0.000156	TRUE
Spelling	ChatGPT with Prompt 1	ChatGPT with Prompt 2	−0.1	0.999997	FALSE
Spelling	ChatGPT with Prompt 1	Google Translate	0.7	0.998836	FALSE
Spelling	ChatGPT with Prompt 2	Google Translate	0.8	0.998267	FALSE
Total_Errors	Bing Translator	ChatGPT with Prompt 1	−30.2	1.75E-05	TRUE
Total_Errors	Bing Translator	ChatGPT with Prompt 2	−30.8	1.25E-05	TRUE
Total_Errors	Bing Translator	Google Translate	−26.8	0.000116	TRUE
Total_Errors	ChatGPT with Prompt 1	ChatGPT with Prompt 2	−0.6	0.999519	FALSE
Total_Errors	ChatGPT with Prompt 1	Google Translate	3.4	0.924502	FALSE
Total_Errors	ChatGPT with Prompt 2	Google Translate	4	0.883903	FALSE

For example, Bing Translator made 30.2 more total errors than ChatGPT with Prompt 1 (p-value = 1.75E-05), and 30.8 more errors than ChatGPT with Prompt 2 (p-value = 1.25E-05). These differences were statistically significant, suggesting that ChatGPT prompts are highly effective in reducing the number of translation errors, particularly compared to Bing Translator.

Fig 3 further illustrates the distribution of these errors across translation tools, providing a clear visualization of how Bing Translator stands out with a much higher frequency of errors, especially in spelling. ChatGPT with Prompt 1 and Prompt 2 consistently produce fewer errors, with Prompt 1 showing a slightly higher rate of errors in grammar than Prompt 2, particularly in the earlier chapters.

To complement the statistical findings, it is essential to examine concrete cases that illustrate how translation errors affect meaning and style. While Tables 11–14 and Fig 3 highlight the quantitative differences among the systems, examples from the corpus reveal how these errors manifest in practice and how they distort culturally or stylistically sensitive content. One such instance is presented below:

**Example:**

**Source text (Chapter 4, excerpt)**: «ئەقەل كەلدۇ قارشى، دىدى دەققەت تەت،

سۆزۈڭ بولسا يىغىلماس، بولۇر ساڭا سەت.»

**Reference translation:** Reason came forth and said, “Take care!” If your words are careless, it will bring you harm.

**ChatGPT with Prompt 1:** Reason has come forth and said, “Be aware,” If your words are careless, they may lead to despair.

**ChatGPT with Prompt 2:** For careless tongues may carve a lasting scar, while noble speech endures like evening star.

**Google Translate:** “It’s the opposite,” he said.Your words are inconsequential, and you are ugly.

**Bing Translator:** “It’s the opposite,” he said. Your words are inconsequential, and you are ugly

In this case, the phrase “دىدى دەققەت تەت” (“said, ‘Be careful!’”) was mistranslated by both Google and Bing as “It’s the opposite”. This represents a polarity reversal because a cautionary imperative was rendered as a contradictory statement. Furthermore, the term “سەت” (meaning “harm” or “misfortune”) was incorrectly translated as “ugly”, which shifts the semantic field from moral consequence to physical appearance. The original conditional structure “if your words are careless, they will bring harm” was lost, since the machine output produced evaluative assertions such as “Your words are inconsequential, and you are ugly.” This distortion removes the intended cause and effect relationship and introduces an inappropriate colloquial register, transforming a didactic maxim into a disparaging remark.

## Discussion

The present study aimed to investigate the performance of various machine translation tools—ChatGPT (with two different prompts), Google Translate, and Bing Translator—across a range of evaluation metrics (BLEU, ROUGE-N, ROUGE-L, METEOR, and BERT) and to analyze the translation errors produced by these tools. The main findings of the study, as discussed in the previous sections, provide important insights into the comparative effectiveness of these tools and the impact of different prompts on ChatGPT’s translation performance. Moreover, the results also shed light on the nature of translation errors, specifically in terms of grammar, spelling, and style.

One of the main findings of this study is that ChatGPT, regardless of the prompt used, consistently outperforms Bing Translator and Google Translate in terms of translation quality, as measured by BLEU, ROUGE, METEOR, and BERT scores. In particular, ChatGPT with Prompt 1 and Prompt 2 yielded higher scores across the evaluation metrics compared to Bing Translator, especially in the BLEU, ROUGE, and METEOR categories (Figs 1–3). Importantly, the results also revealed that Prompt 1 consistently outperformed Prompt 2 in statistical tests, highlighting the effectiveness of concise and direct instructions over interpretive ones when guiding ChatGPT for literary translation. This confirms the role of prompt engineering as a decisive factor in improving LLM-based translations.

Notably, Bing Translator demonstrated a higher frequency of translation errors, particularly in spelling and grammar, which were significantly more pronounced than in the translations produced by ChatGPT and Google Translate. Fig 3 illustrated these differences clearly, showing that Bing Translator had a considerably higher error rate, particularly in the Spelling category, where the number of errors was much greater than that of other tools. In contrast, Google Translate, while not performing at the same level as ChatGPT, produced relatively consistent results with moderate error rates, suggesting its reliability for baseline translation tasks.

The qualitative examples further demonstrated how these errors manifest in practice. For instance, both Google and Bing mistranslated the cautionary imperative “دىدى دەققەت تەت” as “It’s the opposite,” creating a polarity reversal and shifting the semantic field. By comparison, ChatGPT maintained semantic integrity, with Prompt 2 even producing a stylistically enriched rendering. Such cases highlight not only the quantitative superiority of ChatGPT but also its ability to better preserve cultural and stylistic nuances in sensitive contexts.

In comparison with existing literature, these findings corroborate the results of previous studies that have highlighted the superior performance of ChatGPT over traditional machine translation tools like Google Translate and Bing Translator. For instance, recent studies have found that ChatGPT, especially with well-designed prompts, is able to produce translations with a higher degree of fluency and accuracy [52,53]. Moreover, the finding that Bing Translator exhibits a high number of errors, particularly in spelling, aligns with earlier research that has criticized the tool for producing translations that are often marked by spelling inconsistencies [54]. This study also extends prior work by combining automatic evaluation, statistical testing, human expert ratings, and qualitative examples, thus offering a multidimensional understanding of translation performance that goes beyond metric-based comparisons alone.

While the results of this study contribute valuable insights into the comparative performance of machine translation tools, there are also several strengths and limitations that must be considered. A key strength of this study is its use of multiple evaluation metrics—BLEU, ROUGE-N, ROUGE-L, METEOR, and BERT—which provide a comprehensive and multidimensional assessment of translation quality. By utilizing these metrics, the study offers a well-rounded view of how each translation tool performs across different types of evaluations. Additionally, the study’s detailed analysis of translation errors—particularly in terms of grammar, spelling, and style—provides further insights into the specific areas where each tool excels or falls short. The integration of expert ratings confirmed these results, as ChatGPT with Prompt 1 achieved the highest scores in Fidelity, Fluency, and Cultural Fidelity, while Bing Translator scored highest only in exhibiting “machine-like” style.

However, there are limitations to this study that should be acknowledged. One limitation is the relatively small sample size used in the analysis. While ten chapters were selected for evaluation, a larger sample size could provide more robust and generalizable results. Additionally, the study focused on only a limited set of machine translation tools, and it would be valuable to expand the comparison to include other widely used systems, such as DeepL, to further validate the findings. Furthermore, the study’s error analysis is limited to only three categories—grammar, spelling, and style—and future research could explore additional error types or perform a more in-depth qualitative analysis of the errors made by each tool. Another limitation is that post-editing practices, which could significantly alter the usability of raw MT outputs, were not examined. Future studies could investigate the interplay between machine outputs and human editing, especially in low-resource literary contexts.

## Conclusion

This study investigates the performance of machine translation tools—ChatGPT, Google Translate, and Bing Translator—specifically for translating Uighur literature into English. The findings reveal several major contributions. First, ChatGPT with carefully designed prompts consistently outperforms both Google Translate and Bing Translator across automatic evaluation metrics (BLEU, ROUGE, METEOR, and BERT), confirming the effectiveness of prompt engineering in enhancing LLM-based translation. Second, Bing Translator showed significantly higher error rates, especially in spelling and grammar, while Google Translate demonstrated more stable yet moderate performance. Third, error analysis and qualitative examples highlighted that Bing frequently produced polarity reversals and semantic distortions, whereas ChatGPT better preserved semantic integrity and stylistic nuance, particularly under Prompt 2. Finally, expert evaluations corroborated these trends, rating ChatGPT with Prompt 1 highest in Fidelity, Fluency, and Cultural Fidelity.

Beyond these comparative results, this study also makes a methodological contribution by integrating automatic metrics, statistical testing, expert ratings, and qualitative analysis. This multidimensional framework provides a more holistic understanding of translation performance, demonstrating not only how systems score numerically but also how their outputs function in terms of cultural and stylistic adequacy.

While the study focuses on Uighur literature, it provides valuable insights for researchers and translators working with underrepresented languages. The findings highlight both the opportunities and risks of relying on AI tools for literary translation, suggesting that ChatGPT’s adaptability makes it a promising tool when guided by effective prompts, while traditional NMT systems offer stability but less flexibility. However, future research should explore additional translation tools and languages, as well as the impact of post-editing on translation quality. Expanding the dataset and including comparative analyses with other low-resource languages could further validate and extend these findings. This study offers a step toward better understanding how machine translation tools can be optimized for culturally sensitive and literary translation tasks.
